# Prostanoid receptor genes confer poor prognosis in head and neck squamous cell carcinoma via epigenetic inactivation

**DOI:** 10.1186/s12967-020-02214-1

**Published:** 2020-01-21

**Authors:** Kiyoshi Misawa, Masato Mima, Yamada Satoshi, Atsushi Imai, Daiki Mochizuki, Ryuji Ishikawa, Junya Kita, Yuki Yamaguchi, Shiori Endo, Yuki Misawa, Hiroyuki Mineta

**Affiliations:** Department of Otolaryngology/Head and Neck Surgery, Hamamatsu University School of Medicine, 1-20-1 Handayama, Shizuoka, 431-3192 Japan

**Keywords:** Prostanoid receptor genes, GPCRs, TET, Epigenetic markers, Q-MSP

## Abstract

**Background:**

Chronic inflammation is a risk factor for head and neck squamous cell carcinoma (HNSCC) and other diseases. Prostanoid receptors are clearly involved in the development of many types of cancer. However, their role is not simple and is poorly understood in HNSCC.

**Methods:**

Methylation profiles of prostanoid receptor family genes were generated for tumour samples obtained from 274 patients with HNSCC, including 69 hypopharynx, 51 larynx, 79 oral cavity, and 75 oropharynx tumour samples, by quantitative methylation-specific PCR. Promoter methylation was then evaluated with respect to various clinical characteristics and patient survival.

**Results:**

The mean number of methylated genes per sample was 2.05 ± 2.59 (range 0 to 9). Promoters of *PTGDR1*, *PTGDR2*, *PTGER1*, *PTGER2*, *PTGER3*, *PTGER4*, *PTGFR*, *PTGIR*, and *TBXA2R* were methylated in 43.8%, 18.2%, 25.5%, 17.5%, 41.2%, 8.0%, 19.3%, 20.4%, and 11.3% of the samples, respectively. Methylation indices for prostanoid receptor family genes tended to be higher as the number of *TET* methylation events increased. Patients with 5–9 methylated genes had a significantly lower survival rate than that of patients with 0–4 methylated genes (log-rank test, P= 0.007). In multivariate analyses, *PTGDR1* methylation was most highly correlated with recurrence in patients with hypopharyngeal cancer (P = 0.014). A similar correlation was observed for *PTGER4* in patients with laryngeal cancer (P = 0.046). Methylation of the *PTGIR* and *TBXA2R* promoters was positively correlated with recurrence in oropharyngeal cancer (P = 0.028 and P = 0.006, respectively). Moreover, Patients with 5–9 methylated genes were extremely lower of 5hmC levels (P = 0.035) and was correlated with increasing expression of *DNMT3A* and *DNMT3B* (P < 0.05 and P < 0.05, respectively).

**Conclusion:**

We characterised the relationship between the methylation status of prostanoid receptor genes and recurrence in HNSCC. These results provide new perspectives for the development of molecular targeted treatment approaches.

## Background

Clinical and epidemiological evidence suggests that chronic inflammation is a major risk factor for head and neck malignancies [1]. For example, patients with persistent human papilloma virus (HPV) infection, Epstein–Barr virus infection, or chronic inflammation (as observed in individuals with a cigarette smoking habit) face an increased lifetime risk for oropharyngeal cancer, nasopharyngeal cancer, or laryngeal cancer, respectively [2, 3]. Many studies have focused on cytokines and chemokines as mediators connecting chronic inflammation to head and neck cancer, but little is known about the involvement of prostanoid receptors [4]. To improve the survival rate for head and neck cancer, precision medicine approaches, improvements in diagnosis and prognosis, as well as the identification of novel targets and treatment strategies with minimal side effects are required.

G protein-coupled receptors (GPCRs) are the largest class of cell-surface receptors and are involved in many cancers, including head and neck squamous cell carcinoma (HNSCC) [5, 6]. GPCRs are modulated by a variety of endogenous and synthetic ligands and are major drug targets [7]. Key therapeutic applications involving GPCRs include opioid analgesics, antihistamines, anticholinergics, typical and atypical antipsychotics, antimigraine drugs, β2-agonists for asthma, and anti-hypertensives [8]. However, anti-cancer drugs that specifically target GPCRs are not currently available [9]. The prostanoid receptors represent the most notable family of validated pharmacological targets in a variety of diseases, including cancer [10].

Prostanoids derived from arachidonic acid through the cyclooxygenase (COX) pathway are particularly relevant. Prostaglandin H2 (PGH2) is the common cyclic-peroxide intermediate in the biosynthesis of prostanoids derived from arachidonic acid [11]. Prostanoids are a group of lipid mediators that include prostaglandins (PG) and thromboxanes (TX) [12]. Fatty acid COX converts arachidonic acid to PGH2, from which further prostanoids, PGD2, PGE2, PGF2α, PGI2 (prostacyclin), and thromboxane A2 (TXA2), may be enzymatically derived [13]. All nine prostanoid receptor genes [prostaglandin D2 receptors (*PTGDR1* and *PTGDR2*), four prostaglandin E2 receptors (*PTGER1*, *PTGER2*, *PTGER3* and *PTGER4*), the prostaglandin F receptor (*PTGFR*), the prostaglandin I2 receptor (*PTGIR*), and thromboxane A2 receptor (*TBXA2R*)] encode neuropeptide receptors and belong to the GPCR Class Aα subgroup [14]. These nine prostanoid receptor genes have been implicated in the development of multiple types of cancer, but studies of the methylation status of all nine genes and their roles in the prognosis of HNSCC are lacking.

In this study, we provide that associations between the methylation status of nine prostanoid receptor genes and clinicopathological characteristics (e.g., tumour location and recurrence events) were also assessed. To our knowledge, this study is the first to link prostanoid receptor gene methylation to the genesis of HNSCC.

## Methods

### Tumour samples

Surgical HNSCC tumour and matched adjacent non-tumour tissues were obtained from 274 patients who underwent surgical resection at the Department of Otolaryngology/Head and Neck Surgery, Hamamatsu University School of Medicine (Hamamatsu, Shizuoka, Japan). Written informed consent was obtained from individual patients before surgery and the experimental protocol was approved by the Hamamatsu University School of Medicine (date of board approval: October 2, 2015, ethics code: 25-149). The ratio of males to females was 227:47. The mean age was 65.2 years (range, 32 to 90 years). Primary head and neck tumours included 69 hypopharyngeal carcinomas, 51 laryngeal carcinomas, 79 oropharyngeal carcinomas, and 75 oral cavity carcinomas (Additional file 1: Table S1).

### DNA extraction and modification

DNA extraction from fresh tissue was performed using a QIAamp DNA Mini Kit (Qiagen, Hilden, Germany). Sodium bisulphite conversion was performed using the MethylEasy Xceed Rapid DNA Bisulfite Modification Kit (TaKaRa, Tokyo, Japan) following the manufacturer’s protocol.

### Quantitative methylation-specific PCR analysis (Q-MSP)

Aberrant DNA methylation, which often occurs around the transcription start site (TSS) within a CpG island, was evaluated by Q-MSP. The sequences of primers used in this study are shown in Additional file 2: Table S2. Exon one or two and CpG sites within view of the promoter region relative to the TSS are presented in Additional file 3: Figure S1. A standard curve for Q-MSP was constructed by plotting five serially diluted standard solutions of EpiScope Methylated HeLa gDNA (TaKaRa). The normalized methylation value (NMV) was defined as follows: NMV = (prostanoid receptor gene-S/prostanoid receptor gene-FM)/(ACTB-S/ACTB-FM), where prostanoid receptor gene-S and prostanoid receptor gene-FM represent target gene methylation levels in the tumour sample and in the universal methylated DNA control, respectively. ACTB-S and ACTB-FM correspond to β-actin (ACTB) in the sample and the universally methylated DNA, respectively [15].

### Detection of high-risk HPV DNA by PCR

To identify the HPV types, samples were also subjected to PCR using specific primers for HPV types 16, 18, 31, 33, 35, 52, and 58. The prevalence of HPV DNA was examined using the PCR HPV Typing Set (TaKaRa).

### ELISA for 5-hmC quantification

The 5hmC content of genomic DNA was determined with a Quest 5-hmC DNA ELISA Kit (Zymo Research, Irvine, CA, USA), according to the manufacturer’s instructions. The amount of 5-hmC was calculated as a percentage based on a standard curve generated using kit controls.

### RNA extraction and quantitative reverse transcription PCR (qRT-PCR)

Total RNA was isolated using an RNeasy Plus Mini Kit (Qiagen, Hilden, Germany); cDNA was synthesized using a ReverTra Ace qPCR RT Kit (Toyobo, Tokyo, Japan). DNMT3A, DNMT3B, and GAPDH mRNA expression levels were measured via qRT-PCR using SYBR Premix Ex Taq (Takara Bio Inc., Tokyo, Japan), the Takara Thermal Cycler Dice Real Time System TP8000 (Takara Bio Inc.), and the primer sets presented in previously reports [16].

### Data mining in the Cancer Genome Atlas (TCGA)

MethHC (http://methhc.mbc.nctu.edu.tw/php/index.php) was used to extract data from TCGA (available in August 2019). DNA methylation of prostanoid receptor genes was measured using the Illumina Infinium Human Methylation 450 K BeadChip. The methylation score for each CpG site was estimated as the β-value, which ranges from 0 to 1, corresponding to unmethylated and completely methylated DNA, respectively. In addition, RNAseq data of *PTGDR1*, *PTGER1*, *PTGER2*, *PTGER3*, *PTGER4*, *PTGFR*, *PTGIR*, *TBXA2R*, *IL*-*6*, *IL*-*11* and *RANK* were obtained from the TCGA data portal (https://tcga-data.nci.nih.gov/tcga/).

### Data analysis and statistics

A receiver operator characteristic (ROC) curve analysis of target genes was performed using the NMVs for 36 matched paired HNSCC and normal mucosal samples and the Stata/SE 13.0 system (Stata Corporation, College Station, TX, USA). To determine the area under the ROC curve, the true positive rate (Sensitivity) was plotted as a function of the false positive rate (1 − Specificity) for different cut-off points, and the NMV thresholds were calculated for each target gene. Cut-off values showing the greatest accuracy were determined based on sensitivity/specificity, as indicated in Additional file 4: Table S3. The MI was defined as the number of genes with promoter methylation [17]. Student’s *t*-tests were performed to evaluate the associations between clinical variables and MI. Disease-free survival (DFS) was investigated using the Kaplan–Meier method and the log-rank test. The probability of survival can be evaluated by generating a Kaplan–Meier curve. A Cox’s proportional hazards regression analysis that included age (≥ 65 vs. < 65 years), sex, alcohol intake, smoking status, and tumour stage (I–II vs. III–IV) and methylation status was used to identify the multivariate predictive value of prognostic factors. A value of P < 0.05 was considered statistically significant.

## Results

### Characterisation of 36 matched paired head and neck tumour samples and adjacent noncancerous mucosal samples

Promoter hypermethylation of nine prostanoid receptor genes exhibited distinct ROC curve profiles, which clearly differentiate cancer tissues from normal tissues (Additional file 5: Fig. S2). A specimen was classified as methylated when its NMV exceeded 0.161, 0.123, 0.048, 0.161, 0.502, 0.419, 0.368, 0.109, and 0.082 for *PTGDR1*, *PTGDR2*, *PTGER1*, *PTGER2*, *PTGER3*, *PTGER4*, *PTGFR*, *PTGIR,* and *TBXA2R*, respectively (Additional file 4: Table S3). Methylation levels of all prostanoid receptor genes in primary HNSCCs were significantly higher than those in matched paired normal mucosal tissues, except for *PTGER3* (Additional file 6: Fig. S3).

### Analysis of methylation status in HNSCC tissue samples

**Fig. 1:**
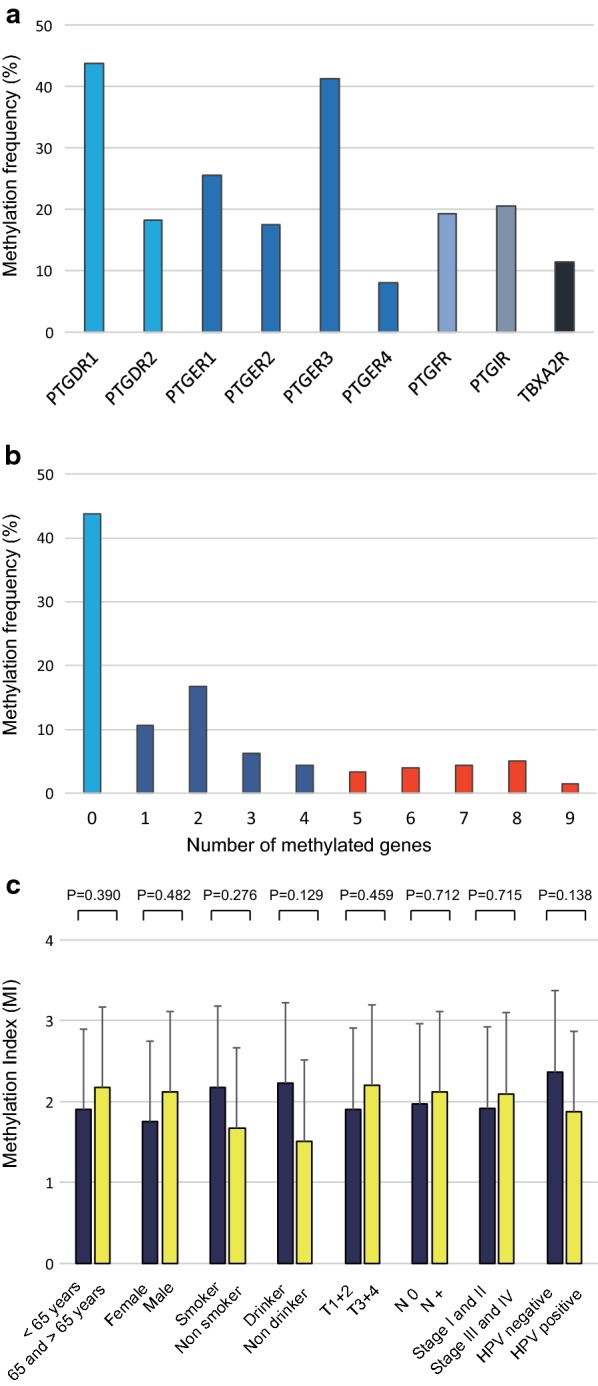
Methylation of nine prostanoid receptor gene promoters in 274 HNSCC samples. **a** Bar graph showing methylation frequencies of the nine genes. **b** Bar graph showing the percentage of tumours with zero to nine methylated target genes. **c** Bar graph showing the MI according to selected clinical parameters. Differences in mean MI for each parameter were determined by Student’s *t*-tests

A Q-MSP analysis of the methylation status of nine prostanoid receptor genes was performed using 274 primary HNSCC samples. The methylation frequencies were as follows: *PTGDR1* (43.8%), *PTGDR2* (18.2%), *PTGER1* (25.5%), *PTGER2* (17.5%), *PTGER3* (41.2%), *PTGER4* (8.0%), *PTGFR* (19.3%), *PTGIR* (20.4%), and *TBXA2R* (11.3%) (Fig. 1a). The average MI per sample was 2.05 ± 2.59 (range 0 to 9) (Fig. 1b). No significant differences in MI were observed with respect to the age at disease onset, sex, alcohol consumption, smoking status, tumour size, lymph node status, clinical stage, or HPV status (Fig. 1c). We analysed the relationships of the methylation status of each prostanoid receptor gene with the clinical features of patients with HNSCC. *PTGFR* methylation was significantly correlated with age at onset (P = 0.043). Methylation levels of *PTGDR2*, *PTGER1,* and *PTGFR* promoters were associated with alcohol exposure (P = 0.042, P = 0.04, and P = 0.049, respectively). There was an association between methylation of the *PTGDR1* and *PTGER3* promoters and HPV status (P = 0.004 and P = 0.005, respectively). We found that the promoter methylation of all prostanoid receptor genes with the exception of *TBXA2R* was associated with recurrence events (Table 1).

**Table 1:** Distribution of methylation status by selected epidemiologic and clinical characteristics

Gene	Methylation status	Characteristics	Age			Gender			Smoking status			Alcohol exposure			
		Overall (%)	< 65	> 65	P^†^	Female	Male	P^†^	Smoker	Non smoker	P^†^	Drinker	Non drinker	P^†^	
PTGDR1	Yes	120 (43.8)	50	70		16	104		97	23		97	23		
	No	154 (56.2)	63	91	1	31	123	0.149	111	43	0.117	112	42	0.152	
PTGDR2	Yes	50 (18.2)	16	34		7	43		42	8		44	6		
	No	224 (81.8)	97	127	0.156	40	184	0.678	166	58	0.199	165	59	0.042*	
PTGER1	Yes	70 (25.5)	30	40		8	62		55	15		60	10		
	No	204 (74.5)	83	121	1	39	165	0.197	153	51	0.628	149	55	0.034*	
PTGER2	Yes	48 (17.5)	18	30		10	38		37	11		39	9		
	No	226 (82.5)	95	131	0.630	37	189	1	171	55	1	170	56	0.457	
PTGER3	Yes	113 (41.2)	49	64		17	96		87	26		86	27		
	No	161 (58.8)	64	97	1	30	131	0.516	121	40	0.775	123	38	1	
PTGER4	Yes	22 (8.0)	6	16		4	18		18	4		18	4		
	No	252 (92.0)	107	145	0.183	43	209	1	190	62	0.610	191	61	0.612	
PTGFR	Yes	53 (19.3)	15	38		8	45		44	9		46	7		
	No	221 (80.7)	98	123	0.043*	39	182	0.839	164	57	0.212	163	58	0.049*	
PTGIR	Yes	56 (20.4)	20	36		8	48		46	10		48	8		
	No	218 (79.6)	93	125	0.366	39	179	0.691	162	56	0.293	161	57	0.078	
TBXA2R	Yes	31 (11.3)	10	21		4	27		27	4		27	4		
	No	243 (88.7)	103	140	0.355	43	200	0.620	181	62	0.179	182	61	0.346	

Gene	Tumor size			Lympho-node status			Stage			HPV status			Recurrence events		
	T1–2	T3–4	P^†^	N0	N+	P^†^	I, II	III, IV	P^†^	Negative	Positive	P^†^	Negative	Positive	P^†^
PTGDR1	54	66		47	73		24	36		105	15		47	68	
	80	74	0.274	61	93	1	96	118	0.557	95	36	0.004*	127	32	< 0.001*
PTGDR2	23	27		16	34		9	51		44	6		22	27	
	111	113	0.755	92	132	0.265	41	173	0.572	156	45	0.118	152	73	0.005*
PTGER1	30	40		27	43		15	45		59	11		29	40	
	104	100	0.269	81	123	0.888	55	159	1	141	40	0.297	145	60	< 0.001*
PTGER2	21	27		19	29		10	50		42	6		19	28	
	113	113	0.525	89	137	1	38	176	1	158	45	0.164	155	72	< 0.001*
PTGER3	52	61		43	70		23	37		99	14		38	72	
	82	79	0.462	65	96	0.708	90	124	0.658	101	37	0.005*	136	28	< 0.001*
PTGER4	11	11		12	10		6	54		20	2		9	13	
	123	129	1	96	156	0.172	16	198	1	180	49	0.266	165	87	0.035*
PTGFR	26	27		19	34		12	48		46	7		22	31	
	108	113	1	89	132	0.639	41	173	1	154	44	0.180	152	69	< 0.001*
PTGIR	28	28		16	40		9	51		48	8		24	32	
	106	112	1	92	126	0.067	47	167	0.280	152	43	0.259	150	68	< 0.001*
TBXA2R	10	21		13	18		7	53		27	4		16	15	
	124	119	0.057	95	148	1	24	190	1	173	47	0.346	158	85	1

* P < 0.05, ^†^Chi squared test

### Comparison of methylation frequencies between nine prostanoid receptor genes and ten-eleven translocation (*TET*) family genes

**Fig. 2:**
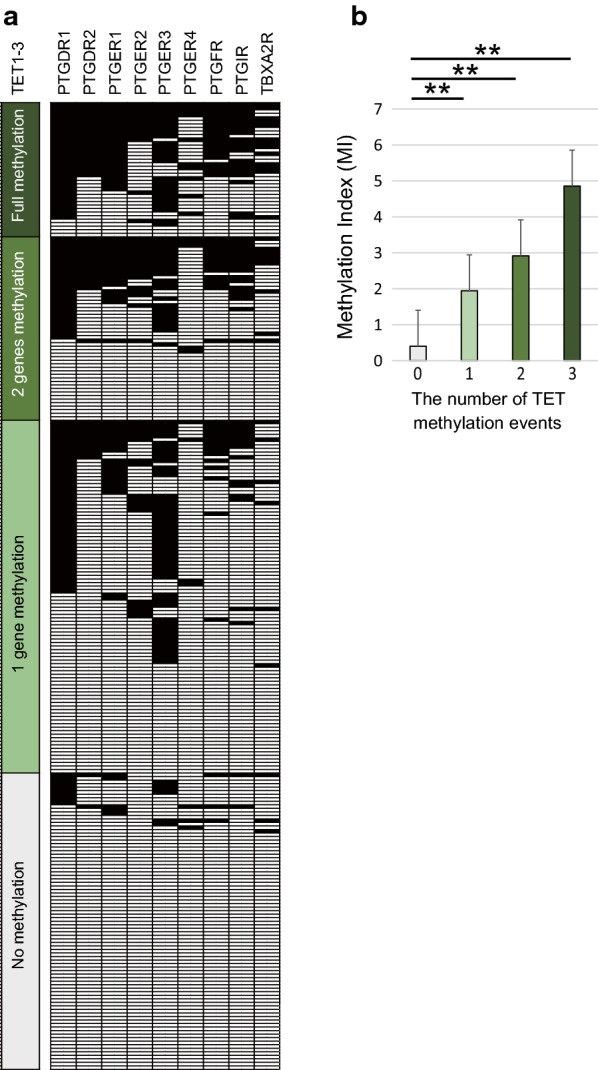
Correlation between promoter methylation levels of the nine prostanoid receptor genes and *TET* family genes in cancer tissues. **a** Distribution of promoter methylation in *TET* family genes and the nine prostanoid receptor genes. Filled boxes indicate the presence of methylation, and open boxes indicate the absence of methylation. **b** Combined analysis of the MI and methylation status of *TET* family genes. The number of methylation events is indicated for hypermethylated *TET* family genes. The mean MI for the different groups were compared using Student’s *t*-tests. **P < 0.001. Data are shown as mean ± SD

Mean differences in the MI of nine prostanoid receptor genes based on *TET* gene methylation events are illustrated in Fig. 2a. The MI was significantly higher in patients with methylation events at all *TET* genes (4.84 ± 2.73), two *TET* gene methylation events (2.92 ± 3.09), and one *TET* gene methylation event (1.94 ± 2.03) than in patients with no *TET* gene methylation events (0.39 ± 1.05; P < 0.01 for all comparisons) (Fig. 2b).

### Survival analysis

**Fig. 3:**
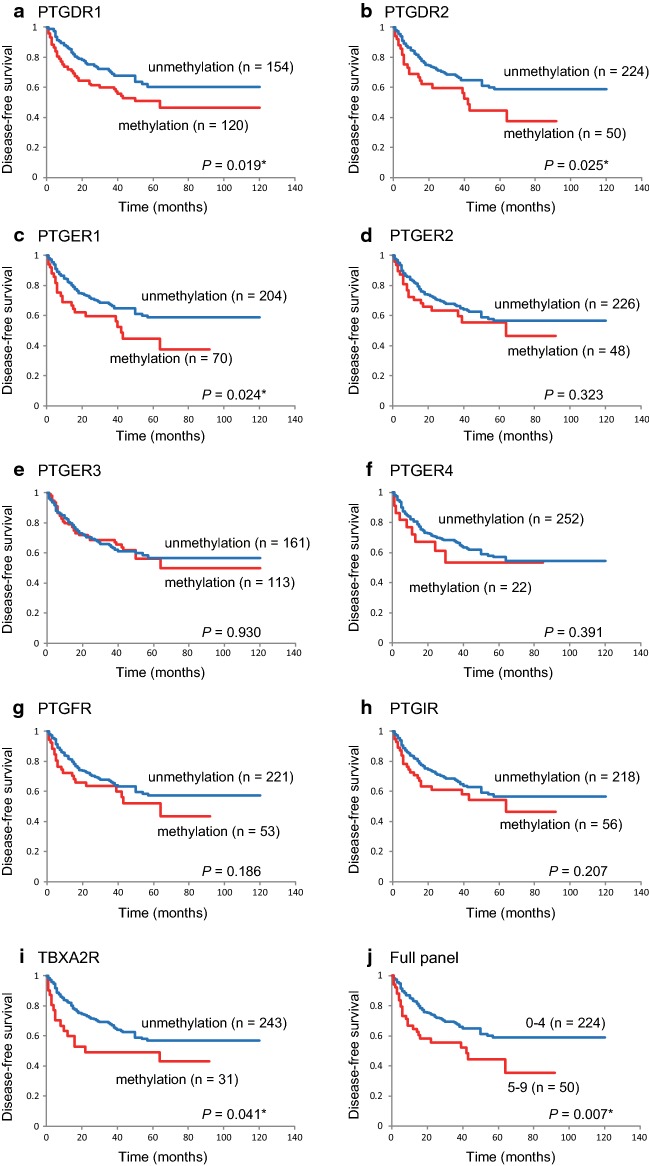
Kaplan–Meier survival curves based on 274 patients with HNSCC according to the methylation status of the nine prostanoid receptor genes. DFS with respect to **a***PTGDR1*, **b***PTGDR2*, **c***PTGER1*, **d***PTGER2*, **e***PTGER3*, **f***PTGER4*, **g***PTGFR*, **h***PTGIR,* and **i***TBXA2R* in the case of methylated (red lines) and unmethylated (blue lines) genes. **j** Combined analysis of the nine genes. Blue line: patients with 0–4 methylated genes; red line: patients with 5–9 methylated genes. *P < 0.05

The results of a Kaplan–Meier analysis of DFS are shown in Fig. 3. DFS did not differ between patients with methylated and unmethylated genes (Fig. 3d–h), with several notable exceptions, i.e., DFS was significantly shorter when the *PTGDR1* (log-rank test, P = 0.019), *PTGDR2* (log-rank test, P = 0.025), *PTGER1* (log-rank test, P = 0.024), and *TBXA2R* (log-rank test, P = 0.041) promoters were methylated (Fig. 3a–c, i). DFS in patients with 5–9 methylated genes was lower than that in the group with 0–4 methylated genes (35.4% versus 59.0%; log-rank test, P = 0.007; Fig. 3j, Additional file 7: Table S4).

### Site-specific analysis of the methylation status

**Fig. 4:**
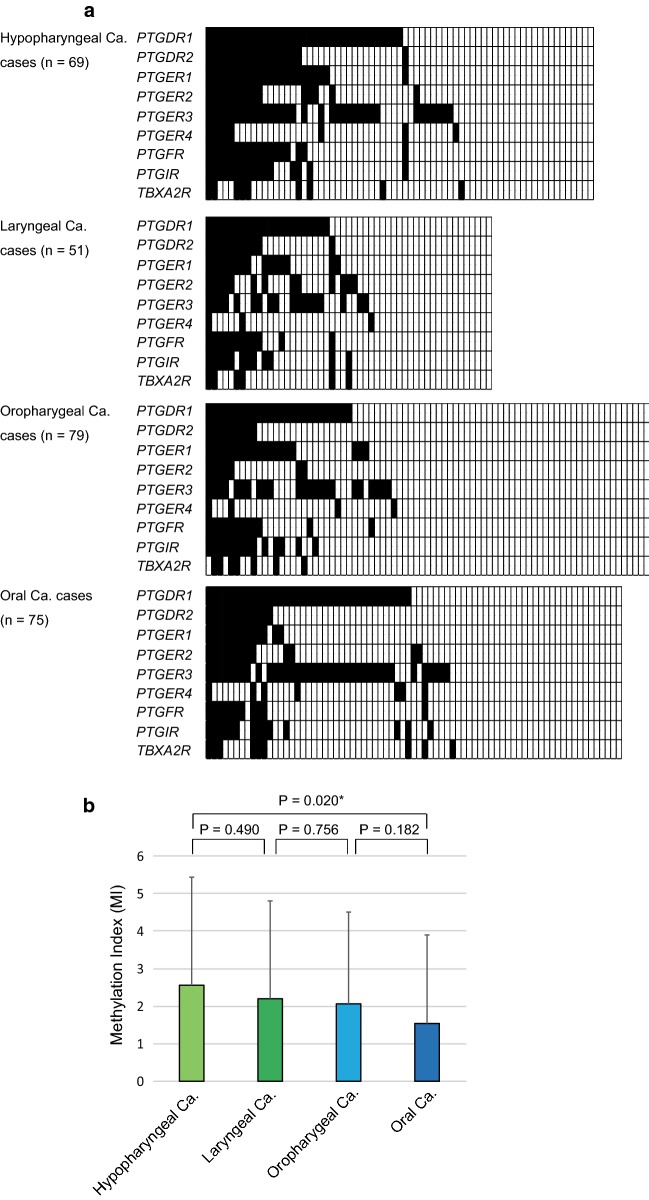
Site-specific methylation frequencies for nine prostanoid receptor genes. **a** Comparison of methylation statuses of the promoters of the nine prostanoid receptor genes in patients with hypopharyngeal, laryngeal, oropharyngeal, or oral cancer. Filled boxes indicate the presence of methylation, and open boxes indicate the absence of methylation. **b** The mean MI values for various groups were compared using Student’s *t*-tests. *P < 0.05

Site-specific methylation frequencies across nine genes for the hypopharynx, larynx, oropharynx, and oral cavity are shown in Fig. 4a. MI levels were significantly higher in patients with hypopharyngeal cancer than in patients with oral cavity cancer (P = 0.020) (Fig. 4b). Among 69 cases with hypopharyngeal cancer, the DFS rate in those with *PTGDR1* methylation was similar to that in the unmethylated group (log-rank test, P = 0.011; Additional file 8: Fig. S4A). Patients with laryngeal cancer and methylated *PTGER4* promoters had a relatively short DFS (log-rank test, P = 0.020; Additional file 8: Fig. S4B). Additional analysis including only patients with oropharyngeal cancer (n = 79) revealed a shorter DFS for methylated than for unmethylated *PTGIR* and *TBXA2R* (log-rank test, P = 0.003 and P = 0.009, respectively; Additional file 8: Fig. S4C, D).

### Stratification analysis

**Fig. 5:**
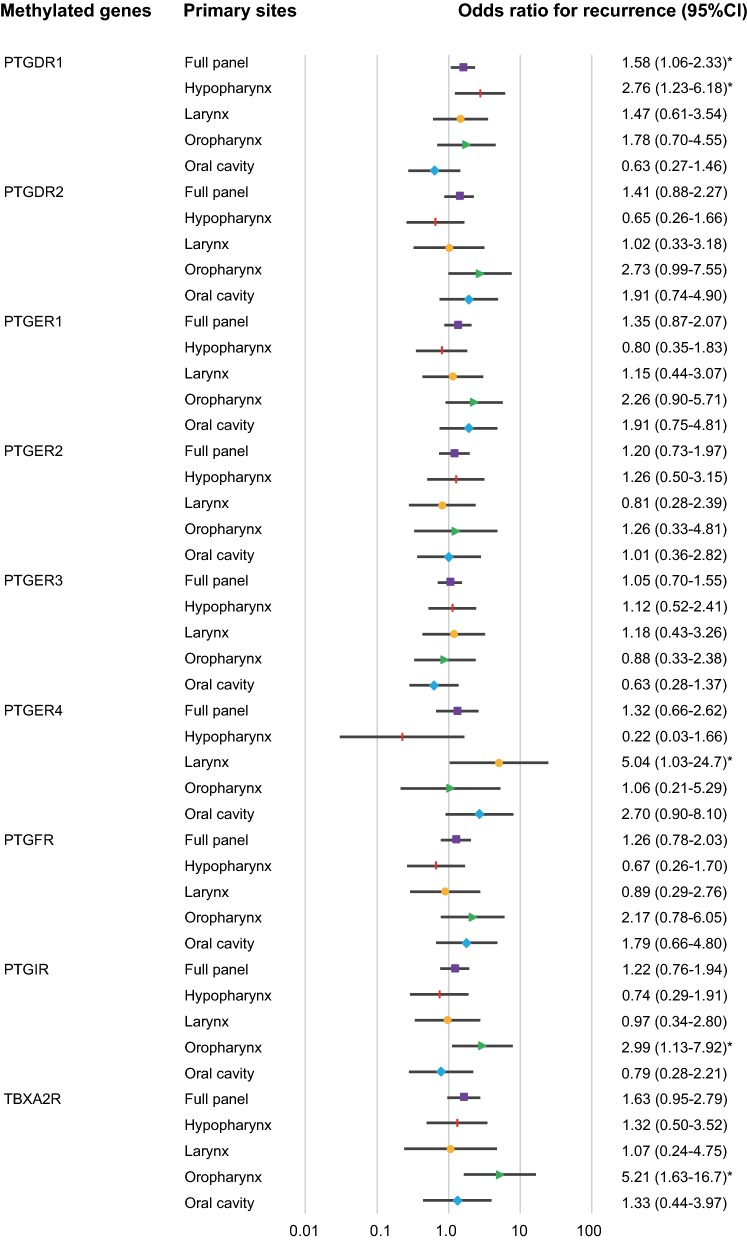
Risk of recurrence based on gene methylation in tumours of different origins. Odds ratios for recurrence were determined using a Cox proportional hazards model adjusted for age (≥ 65 vs. < 65 years), sex, smoking status, alcohol intake, and tumour stage (I–II vs. III–IV). *CI* confidence interval. *P < 0.05

The relation between the methylation status and risk of recurrence was analysed by a multivariate analysis using a Cox proportional hazards regression model adjusted for age, sex, smoking status, alcohol consumption, and clinical stage. For 274 patients with *PTGDR1* promoter methylation, the adjusted odds ratio (OR) for recurrence was 1.58 [95% confidence interval (CI) 1.06–2.33, P = 0.023]. In patients with hypopharyngeal cancer, *PTGDR1* promoter methylation was significantly associated with recurrence (OR = 2.76, 95% CI 1.23–6.18, P = 0.014). For patients with laryngeal cancer with a methylated *PTGER4* promoter, the OR was 5.04 (95% CI 1.03–24.70; P = 0.046). Methylation statuses of the *PTGIR* and *TBXA2R* promoters were positively correlated with recurrence in patients with oropharyngeal cancer (OR, 2.99; 95% CI 1.13–7.92; P = 0.028 and OR, 5.21; 95% CI 1.63–16.67; P = 0.006, respectively) (Fig. 5).

### Comparison of methylation frequencies between nine prostanoid receptor genes and other epigenetic factors

The 5-hmC level showed the greatest decrease when prostanoid receptor genes were 9 to 5 methylation events (P = 0.035; Additional file 9: Fig. S5A). The *DNMT3A* mRNA expression levels were significantly higher in groups with 9 to 5 methylation and 4 to 1 methylation (P = 0.026 and P = 0.029, respectively; Additional file 9: Fig. S5B). The *DNMT3B* mRNA expression was significantly correlated with both 9 to 5 methylation and 4 to 1 methylation (P = 0.013 and P = 0.020, respectively; Additional file 9: Fig. S5C).

### Analysis of methylation and expression data from TCGA

The methylation status of prostanoid receptor gene promoters was estimated in an additional 516 HNSCC samples and 50 normal samples from TCGA. The average β-values (indicating promoter methylation) for the nine genes were significantly higher in the HNSCC samples than in the normal samples (P < 0.05), except for *PTGER4* and *TBXA2R* (Additional file 10: Fig. S6). The expression of prostanoid receptor genes were significantly higher in *IL*-*6* mRNA high expression group (P < 0.05), except for *PTGER1* gene. *IL*-*11* expression was positively correlated with *PTGDR1* and *TBXA2R* expression (P = 0.001 and P < 0.001, respectively). Expression of *RANKL* was concurrently associated with all prostanoid receptor genes expression (Additional file 11: Table S5).

## Discussion

Epigenetic modifications of prostanoid receptor genes may contribute to tumour development and recurrence. We analysed the methylation statuses of genes encoding neuropeptide GPCRs in 274 HNSCCs originating in the hypopharynx, larynx, oropharynx, or oral cavity. We also compared the methylation status of genes in matched HNSCC and normal samples using data from TCGA. We found that the aberrant methylation of some prostanoid receptor gene promoters is positively correlated with recurrence in patients with HNSCC. In addition, a site-specific analysis revealed that abnormal CpG island hypermethylation was independently associated with aggressive clinical behaviour.

Cancer may be related to chronic inflammation associated with persistent infections, immune-mediated damage, or prolonged exposure to irritants. Genetic and epigenetic alterations underlying carcinogenesis inevitably modify tissue homeostasis and may induce a chronic inflammatory response. Over 20 years ago, non-steroidal anti-inflammatory drugs (NSAIDs) were reported to have anti-colon cancer effects [18]. NSAIDs, which are potent inhibitors of COX, exert chemopreventive effects in cancer development [19]. Numerous epidemiological studies have shown that the regular intake of the NSAID aspirin, an inhibitor of COXs, substantially reduces both the incidence and progression of several prevalent cancers [20]. Abundant epidemiological and preclinical/clinical studies have demonstrated that celecoxib, a specific COX-2 inhibitor, is related to the suppression of cancer cell proliferation and a decrease in cancer incidence [21]. COX-1 is constitutively expressed in many tissues and regulates basal levels of prostaglandins [22]. COX-2 is responsible for the release of prostaglandins after an infection, injury, or in cancer development [23]. In HNSCC, IL-1 released by tumour cells plays a key role in inducing the expression of COX-2 in fibroblasts [11]. Secretion of TGF-β and PGE2 by the HNSCC cells was increased following EGFR inhibition [24]. IL-6, TNF-a and PGE2 produced by primary oral keratinocytes and carcinoma cells may induce oral mucosal inflammation [25]. Recent studies continue to support the prostanoid pathway as a promising target for future HNSCC therapies.

Prostanoids, including PGD2, PGE2, PGF2α, PGI2, and TXA2, activate nine GPCRs, namely *PTGDR1*, *PTGDR2*, *PTGER1*, *PTGER2*, *PTGER3*, *PTGER4*, *PTGFR*, *PTGIR,* and *TBXA2R*. *PTGDR1* downregulation by DNA hypermethylation is correlated with colorectal cancer development [26, 27]. *PTGDR1* methylation from cervical scraping is a promising marker of endometrial cancer and ovarian cancer [28]. *PTGER1* shows a strong association with DNA methylation in non-functioning adrenocortical adenoma [29]. The expression of *PTGER2* is often silenced in neuroblastoma cell lines by epigenetic mechanisms [30]. Increased DNA methylation of *PTGER2* is associated with the progression of neuroblastomas [30], non-small cell lung cancer [31], and cervical cancer tissue [32], suggesting that the aberrant methylation of this gene regulates cell proliferation. Cebola et al. detected *PTGER3* and *PTGFR* hypermethylation in a high proportion of colorectal cancer cases, suggesting that DNA methylation is an important mechanism involved in the deregulation of this pathway [33]. The measurement of *PTGER4* methylation in plasma DNA obtained by minimally invasive sampling can be used to detect malignant lung disease [34]. The loss of methylation and activation of *PTGER4* can explain the acquisition of endocrine therapy resistance and is a therapeutic target for breast cancer [35].

Cancers of the upper aerodigestive tract account for the majority of squamous cell carcinomas, which develop in the epithelial linings of the oral cavity, pharynx, and larynx [36]. There are several subclassifications based on anatomic location, aetiology, and molecular findings [37]. Head and neck cancers arise from a multistep process involving the accumulation of genetic and epigenetic alterations [38]. DNA methylation is a frequent and key epigenetic mechanism underlying the regulation of processes associated with neoplastic transformation [38]. Recently, TET proteins have been identified as important epigenetic modifiers via their dioxygenase activity [39]. TET expression and activity are inhibited by genetic mutations and high methylation of their own promoters [40]. Our data showed that increased DNA methylation of TET genes is correlated with the accumulation of prostanoid receptor genes with aberrant methylation; this may be a meaningful DNA methylation event in HNSCC progression. Furthermore, the groups of high MI were extremely low of 5hmC levels and was correlated with increasing expression of *DNMT3A* and *DNMT3B*. To our knowledge, our study is the first to suggest that *PTGDR1, PTGER4, PTGIR,* and *TBXA2R* methylation is associated with worse DFS and may be a critical event in hypopharyngeal cancers, laryngeal cancers, oropharyngeal cancers, and oral cancers, respectively. However, our results obtained from human specimens and high-throughput profiling platforms may be susceptible to measurement bias from various sources. The current study continues to support the prostanoid receptor as a promising target for future HNSCC therapies.

We systematically evaluated the methylation status of the promoters of nine prostanoid receptor genes and the relationship between methylation and clinical characteristics in HNSCC samples. We identified a novel prognostic biomarker based on promoter DNA methylation changes in operable HNSCC to identify patients at high risk of recurrence and provide complementary epigenetic characterization of this tumour type. Collectively, these data demonstrate the functional roles of the epigenetic regulation of prostanoid receptors and show that these loci are potential targets for epigenetic therapies for inflammatory disorders, such as HNSCC.

## Conclusion

We determined the relationship between the methylation status of prostanoid receptor genes and recurrence in HNSCC, providing new perspectives for the development of molecular targeted therapeutic approaches. To our knowledge, our study provides the first evidence for an association between *PTGDR1*, *PTGER4*, *PTGIR*, and *TBXA2R* methylation and worse survival in hypopharyngeal cancers, laryngeal cancers, oropharyngeal cancers, and oral cancers, respectively. This study involving human specimens and high-throughput profiling platforms may be susceptible to measurement bias from various sources; accordingly the use of methylation markers in clinical practice requires further testing in prospective studies with larger HNSCC cohorts.

## Supplementary information

**Additional file 1: Table S1.** Baseline characteristics of the HNSCC patients.

**Additional file 2: Table S2.** Q-MSP primer list.

**Additional file 3: Fig. S1.** Schematic representation of *PTGDR1*, *PTGDR2*, *PTGER1*, *PTGER2*, *PTGER3*, *PTGER4*, *PTGFR*, *PTGIR* and *TBXA2R* genes. CpG sites are within the expanded views of the promoter region. Vertical lines, individual CpG sites; black box, relative location of the primers used for Q-MSP; bent arrow, translation start site (ATG).

**Additional file 4: Table S3.** Results of the ROC curve analysis, the sensitivity, specificity, and cutoff value.

**Additional file 5: Fig. S2.** Receiver operating characteristic (ROC) curves for the methylation markers in cancer tissue versus adjacent normal mucosal tissue. Based on the ROC curve analysis, Area Under Curves (AUCs) are 0.6767 for *PTGDR1* (A), 0.6265 for *PTGDR2* (B), 0.6574 for *PTGER1* (C), 0.6154 for *PTGER2* (D), 0.4784 for *PTGER3* (E), 0.5405 for *PTGER4* (F), 0.6289 for *PTGFR* (G), 0.6736 for *PTGIR* (H) and 0.6605 for *TBXA2R* (I).

**Additional file 6: Fig. S3.** Hypermethylation patterns in 36 matched pairs of head and neck tumors and adjacent normal mucosal tissues. The NMVs for the *PTGDR1* (A), *PTGDR2* (B), *PTGER1* (C), *PTGER2* (D), *PTGER3* (E), *PTGER4* (F), *PTGFR* (G), *PTGIR* (H) and *TBXA2R* (I) promoters were significantly higher in head and neck tumor tissues (T) than in paired adjacent normal mucosal tissue (N). The differences were significant as determined by the Student’s t‑test. *P < 0.05.

**Additional file 7: Table S4.** Results of log-rank tests for effect of number of methylated genes on disease free survival in 274 HNSCC.

**Additional file 8: Fig. S4.** Kaplan–Meier survival curves. Kaplan–Meier survival curves for *PTGDR1* in (A) patients with hypopharyngeal cancer (n = 69), for *PTGER4* in (B) patients with laryngeal cancer (n = 51), and for *PTGIR* and *TBXA2R* in (C and D) patients with oropharyngeal cancer (n = 79). The log-rank test was used to compare the survival times between patients with methylated (red lines) and unmethylated (blue lines) genes. *P < 0.05.

**Additional file 9: Fig. S5.** Comparison of methylation frequencies between nine prostanoid receptor genes and other epigenetic factors. (A) 5hmC levels, (B) DNMT3A mRNA levels, (C) DNMT3B mRNA levels. *P < 0.05. The data are shown as the mean ± SE.

**Additional file 10: Fig. S6.** Methylation status of the five neuropeptide receptor genes in HNSCC and normal samples in TCGA database. The methylation data for *PTGDR1*, *PTGDR2*, *PTGER1*, *PTGER2*, *PTGER3*, *PTGER4*, *PTGFR*, *PTGIR* and *TBXA2R* in HNSCC and normal samples were collected from TCGA database. *P < 0.05.

**Additional file 11: Table S5.** Distribution of expression levels in TCGA cohort of HNSCC.

## Data Availability

The datasets during and/or analyzed during the current study available from the corresponding author on reasonable request.

## Abbreviations

COX: cyclooxygenase
DFS: disease-free survival
GPCRs: G protein-coupled receptors
HNSCC: head and neck squamous cell carcinoma
HPV: human papilloma virus
NMV: normalized methylation value
NSAIDs: non-steroidal anti-inflammatory drugs
PG: prostaglandins
PTGDR: prostaglandin D2 receptors
PTGER: prostaglandin E2 receptors
PTGFR: prostaglandin F receptor
PTGIR: prostaglandin I2 receptor
Q-MSP: Quantitative methylation-specific PCR
ROC: receiver-operator characteristic
TCGA: Cancer Genome Atlas
TBXA2R: thromboxane A2 receptor
TET: ten-eleven translocation
TSS: transcription start site
TX: thromboxanes
TXA2: thromboxane A2

## References

[1] Tezal M (2012). Interaction between chronic inflammation and oral HPV infection in the etiology of head and neck cancers. Int J Otolaryngol.

[2] Guidry JT, Scott RS (2017). The interaction between human papillomavirus and other viruses. Virus Res.

[3] Stell PM (1972). Smoking and laryngeal cancer. Lancet.

[4] Wang D, DuBois RN (2018). Role of prostanoids in gastrointestinal cancer. J Clin Invest.

[5] Bar-Shavit R, Maoz M, Kancharla A, Nag JK, Agranovich D, Grisaru-Granovsky S, Uziely B (2016). G protein-coupled receptors in cancer. Int J Mol Sci..

[6] Misawa K, Imai A, Mochizuki D, Misawa Y, Endo S, Hosokawa S, Ishikawa R, Mima M, Shinmura K, Kanazawa T (2017). Genes encoding neuropeptide receptors are epigenetic markers in patients with head and neck cancer: a site-specific analysis. Oncotarget.

[7] Wacker D, Stevens RC, Roth BL (2017). How ligands illuminate GPCR molecular pharmacology. Cell.

[8] Heng BC, Aubel D, Fussenegger M (2014). G protein-coupled receptors revisited: therapeutic applications inspired by synthetic biology. Annu Rev Pharmacol Toxicol.

[9] Arakaki AKS, Pan WA, Trejo J (2018). GPCRs in cancer: protease-activated receptors, endocytic adaptors and signaling. Int J Mol Sci..

[10] Majumder M, Nandi P, Omar A, Ugwuagbo KC, Lala PK (2018). EP4 as a therapeutic target for aggressive human breast cancer. Int J Mol Sci..

[11] Alcolea S, Anton R, Camacho M, Soler M, Alfranca A, Aviles-Jurado FX, Redondo JM, Quer M, Leon X, Vila L (2012). Interaction between head and neck squamous cell carcinoma cells and fibroblasts in the biosynthesis of PGE2. J Lipid Res.

[12] Tsuboi K, Sugimoto Y, Ichikawa A (2002). Prostanoid receptor subtypes. Prostaglandins Other Lipid Mediat.

[13] Honda T, Kabashima K (2015). Prostanoids in allergy. Allergol Int.

[14] Narumiya S, Sugimoto Y, Ushikubi F (1999). Prostanoid receptors: structures, properties, and functions. Physiol Rev.

[15] Misawa K, Mima M, Imai A, Mochizuki D, Misawa Y, Endo S, Ishikawa R, Kanazawa T, Mineta H (2018). The neuropeptide genes SST, TAC1, HCRT, NPY, and GAL are powerful epigenetic biomarkers in head and neck cancer: a site-specific analysis. Clin Epigenetics.

[16] Mochizuki D, Misawa Y, Kawasaki H, Imai A, Endo S, Mima M, Yamada S, Nakagawa T, Kanazawa T, Misawa K (2018). Aberrant epigenetic regulation in head and neck cancer due to distinct EZH2 overexpression and DNA hypermethylation. Int J Mol Sci..

[17] Imai A, Mochizuki D, Misawa Y, Nakagawa T, Endo S, Mima M, Yamada S, Kawasaki H, Kanazawa T, Misawa K (2019). SALL2 is a novel prognostic methylation marker in patients with oral squamous carcinomas: associations with SALL1 and SALL3 methylation status. DNA Cell Biol.

[18] Kune GA, Kune S, Watson LF (1988). Colorectal cancer risk, chronic illnesses, operations, and medications: case control results from the Melbourne Colorectal Cancer Study. Cancer Res.

[19] Gurpinar E, Grizzle WE, Piazza GA (2013). COX-independent mechanisms of cancer chemoprevention by anti-inflammatory drugs. Front Oncol.

[20] Wong RSY (2019). Role of nonsteroidal anti-inflammatory drugs (NSAIDs) in cancer prevention and cancer promotion. Adv Pharmacol Sci.

[21] Nasry WHS, Rodriguez-Lecompte JC, Martin CK (2018). Role of COX-2/PGE2 mediated inflammation in oral squamous cell carcinoma. Cancers..

[22] Ornelas A, Zacharias-Millward N, Menter DG, Davis JS, Lichtenberger L, Hawke D, Hawk E, Vilar E, Bhattacharya P, Millward S (2017). Beyond COX-1: the effects of aspirin on platelet biology and potential mechanisms of chemoprevention. Cancer Metastasis Rev.

[23] Ricciotti E, FitzGerald GA (2011). Prostaglandins and inflammation. Arterioscler Thromb Vasc Biol.

[24] Kumai T, Oikawa K, Aoki N, Kimura S, Harabuchi Y, Celis E, Kobayashi H (2014). Tumor-derived TGF-beta and prostaglandin E2 attenuate anti-tumor immune responses in head and neck squamous cell carcinoma treated with EGFR inhibitor. J Transl Med.

[25] Jeng JH, Wang YJ, Chiang BL, Lee PH, Chan CP, Ho YS, Wang TM, Lee JJ, Hahn LJ, Chang MC (2003). Roles of keratinocyte inflammation in oral cancer: regulating the prostaglandin E2, interleukin-6 and TNF-alpha production of oral epithelial cells by areca nut extract and arecoline. Carcinogenesis.

[26] Kalmar A, Peterfia B, Hollosi P, Galamb O, Spisak S, Wichmann B, Bodor A, Toth K, Patai AV, Valcz G (2015). DNA hypermethylation and decreased mRNA expression of MAL, PRIMA1, PTGDR and SFRP1 in colorectal adenoma and cancer. BMC Cancer.

[27] Ooki A, Maleki Z, Tsay JJ, Goparaju C, Brait M, Turaga N, Nam HS, Rom WN, Pass HI, Sidransky D (2017). A panel of novel detection and prognostic methylated DNA markers in primary non-small cell lung cancer and serum DNA. Clin Cancer Res.

[28] Chang CC, Wang HC, Liao YP, Chen YC, Weng YC, Yu MH, Lai HC (2018). The feasibility of detecting endometrial and ovarian cancer using DNA methylation biomarkers in cervical scrapings. J Gynecol Oncol.

[29] Itcho K, Oki K, Kobuke K, Yoshii Y, Ohno H, Yoneda M, Hattori N (2018). Aberrant G protein-receptor expression is associated with DNA methylation in aldosterone-producing adenoma. Mol Cell Endocrinol.

[30] Sugino Y, Misawa A, Inoue J, Kitagawa M, Hosoi H, Sugimoto T, Imoto I, Inazawa J (2007). Epigenetic silencing of prostaglandin E receptor 2 (PTGER2) is associated with progression of neuroblastomas. Oncogene.

[31] Tian L, Suzuki M, Nakajima T, Kubo R, Sekine Y, Shibuya K, Hiroshima K, Nakatani Y, Fujisawa T, Yoshino I (2008). Clinical significance of aberrant methylation of prostaglandin E receptor 2 (PTGER2) in nonsmall cell lung cancer: association with prognosis, PTGER2 expression, and epidermal growth factor receptor mutation. Cancer.

[32] Farkas SA, Milutin-Gasperov N, Grce M, Nilsson TK (2013). Genome-wide DNA methylation assay reveals novel candidate biomarker genes in cervical cancer. Epigenetics.

[33] Cebola I, Custodio J, Munoz M, Diez-Villanueva A, Pare L, Prieto P, Ausso S, Coll-Mulet L, Bosca L, Moreno V (2015). Epigenetics override pro-inflammatory PTGS transcriptomic signature towards selective hyperactivation of PGE2 in colorectal cancer. Clin Epigenetics.

[34] Weiss G, Schlegel A, Kottwitz D, Konig T, Tetzner R (2017). Validation of the SHOX2/PTGER4 DNA methylation marker panel for plasma-based discrimination between patients with malignant and nonmalignant lung disease. J Thorac Oncol.

[35] Hiken JF, McDonald JI, Decker KF, Sanchez C, Hoog J, VanderKraats ND, Jung KL, Akinhanmi M, Rois LE, Ellis MJ (2017). Epigenetic activation of the prostaglandin receptor EP4 promotes resistance to endocrine therapy for breast cancer. Oncogene.

[36] Ausoni S, Boscolo-Rizzo P, Singh B, Da Mosto MC, Spinato G, Tirelli G, Spinato R, Azzarello G (2016). Targeting cellular and molecular drivers of head and neck squamous cell carcinoma: current options and emerging perspectives. Cancer Metastasis Rev.

[37] Klussmann JP (2017). Head and neck cancer—new insights into a heterogeneous disease. Oncol Res Treat.

[38] Takeshima H, Ushijima T (2019). Accumulation of genetic and epigenetic alterations in normal cells and cancer risk. NPJ Precis Oncol.

[39] Misawa K, Imai A, Mochizuki D, Mima M, Endo S, Misawa Y, Kanazawa T, Mineta H (2018). Association of TET3 epigenetic inactivation with head and neck cancer. Oncotarget.

[40] Ichimura N, Shinjo K, An B, Shimizu Y, Yamao K, Ohka F, Katsushima K, Hatanaka A, Tojo M, Yamamoto E (2015). Aberrant TET1 methylation closely associated with CpG island methylator phenotype in colorectal cancer. Cancer Prev Res.

