# Supplementary material for: Prostanoid receptor genes confer poor prognosis in head and neck squamous cell carcinoma via epigenetic inactivation
Source: J Transl Med. 2020 Jan 21;18:31. doi: 10.1186/s12967-020-02214-1 (PMC6977280; doi:10.1186/s12967-020-02214-1)
Supplement: Supplementary file 1 — Additional file 1: Table S1. Baseline characteristics of the HNSCC patients. [file 12967_2020_2214_MOESM1_ESM.pdf]

Additional file 1: Table S1. Baseline characteristics of the HNSCC patients (n = 274)

Characteristic		No. of patients (%)
Age		
	< 65	113 (41.2%)
	> 65	161 (58.8%)
Sex		
	Female	47 (17.2%)
	Male	227 (82.8%)
Tumor location		
	Hypopharynx	69 (25.2%)
	Larynx	51 (18.6%)
	Oropharynx	79 (28.8%)
	Oral cavity	75 (27.4%)
Smoking status		
	smoker	208 (75.9%)
	non smoker	66 (24.1%)
Alcohol exposure		
	drinker	209 (76.3%)
	non drinker	65 (23.7%)
HPV status		
	negative	223 (81.4%)
	positive	51 (18.6%)
Tumor size		
	T1	33 (12.0%)
	T2	101 (36.9%)
	T3	51 (18.6%)
	T4	89 (32.5%)
Lympho-node status		
	N0	108 (39.4%)
	N+	166 (60.6%)
Stage		
	I	22 (8.0%)
	II	38 (13.9%)
	III	50 (18.2%)
	IV	164 (59.9%)
Recurrence events		
	positive	174 (63.5%)
	negative	100 (36.5%)
