# Supplementary material for: Prostanoid receptor genes confer poor prognosis in head and neck squamous cell carcinoma via epigenetic inactivation
Source: J Transl Med. 2020 Jan 21;18:31. doi: 10.1186/s12967-020-02214-1 (PMC6977280; doi:10.1186/s12967-020-02214-1)
Supplement: Supplementary file 2 — Additional file 2: Table S2. Q-MSP primer list. [file 12967_2020_2214_MOESM2_ESM.pdf]

Additional file 2: Table S2. Q-MSP primer list.

Gene	Forward primer 5’-3’	Reverse primer 5’-3’	Base pairs
PTGDR1	TTTCGTACGTTATGAAGTCGT	CGCCCATCACCGCCGAATTAC	81
PTGDR2	GTTACGTGTAATTCGCGGTAG	CAAACTCACGACCGCGTAACT	105
PTGER1	GGGCGAGGCGATTATATGCG	ATTAAACACGACGCCCAACGT	115
PTGER2	GTAGGCGCGGGAGTTTCGAG	CCGACGACGACTTACCCTAA	90
PTGER3	AGTGAGTTTTGGCGTCGTCG	CGTTTACCGCGACTAAAACTA	102
PTGER4	AGATGTTGCGGTTCGCGGAT	ACCACTAAAACCGACGACCGT	90
PTGFR	TCGCGTTAAGGGAACGAGTG	GCCGCTACCTCAAATTCCACCG	103
PTGIR	ATGGCGGATTCGTGTAGGAA	CCGATCGCCGTACGCTCAAA	130
TBXA2R	AGAGAGCGAGTCGCGGGTTG	AGC CCC TAA AAT CGC GAC CTA	90
TET1	ATCGGCGCGAGTTGGAAAGTT	GACCCCAACTCACCGCTAACCG	103
TET2	CGCGGGTAACGGGATTTAAAG	GTACCCTCGCTCTAACCCCCG	123
TET3	CGAGGGGGTGGAGATGGTCGAAAGAAAC	CGTACGACGATTAATACAACT	108
ACTB	TGGTGATGGAGGAGGTTTAGAAGT	AACCAATAAAACCTACTCCTCCCTTAA	133
