# Supplementary figures and images for: Prostanoid receptor genes confer poor prognosis in head and neck squamous cell carcinoma via epigenetic inactivation
Source: J Transl Med. 2020 Jan 21;18:31. doi: 10.1186/s12967-020-02214-1 (PMC6977280; doi:10.1186/s12967-020-02214-1)

Figure S1 Kiyoshi Misawa

A PTGDR1

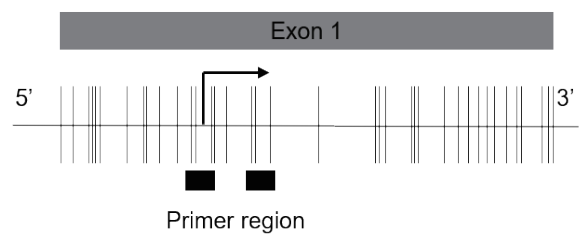

B PTGDR2

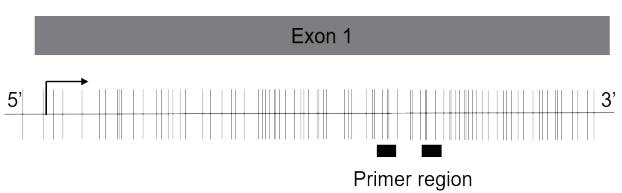

C PTGER1

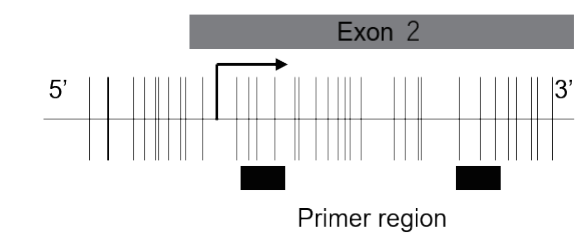

D PTGER2

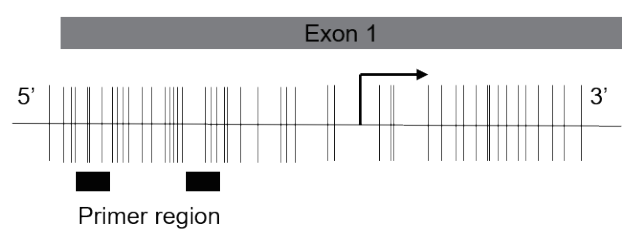

E PTGER3

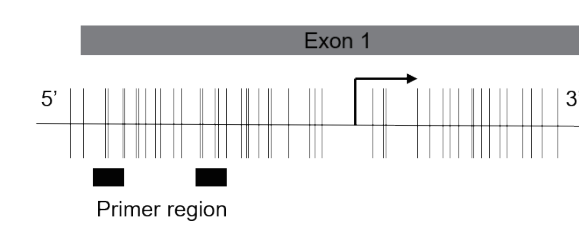

F PTGER4

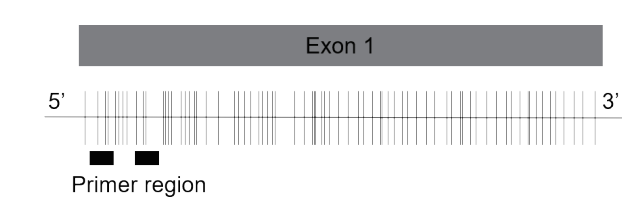

G PTGFR

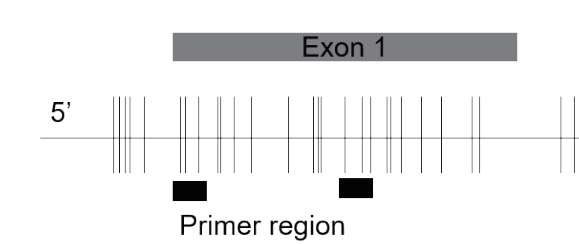

H PTGIR

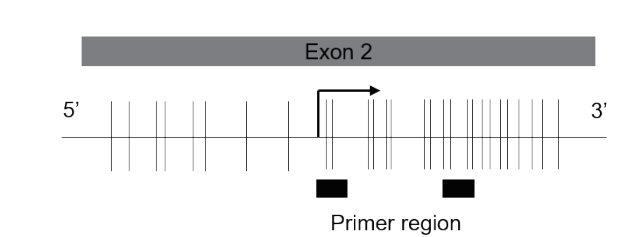

I TBXA2R

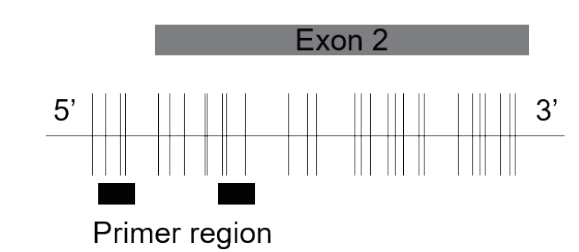

Supplement: Supplementary file 3 — Additional file 3: Fig. S1. Schematic representation of PTGDR1, PTGDR2, PTGER1, PTGER2, PTGER3, PTGER4, PTGFR, PTGIR and TBXA2R genes. CpG sites are within the expanded views of the promoter region. Vertical lines, individual CpG sites; black box, relative location of the primers used for Q-MSP; bent arrow, translation start site (ATG). [file 12967_2020_2214_MOESM3_ESM.pdf]

Figure S2 Kiyoshi Misawa

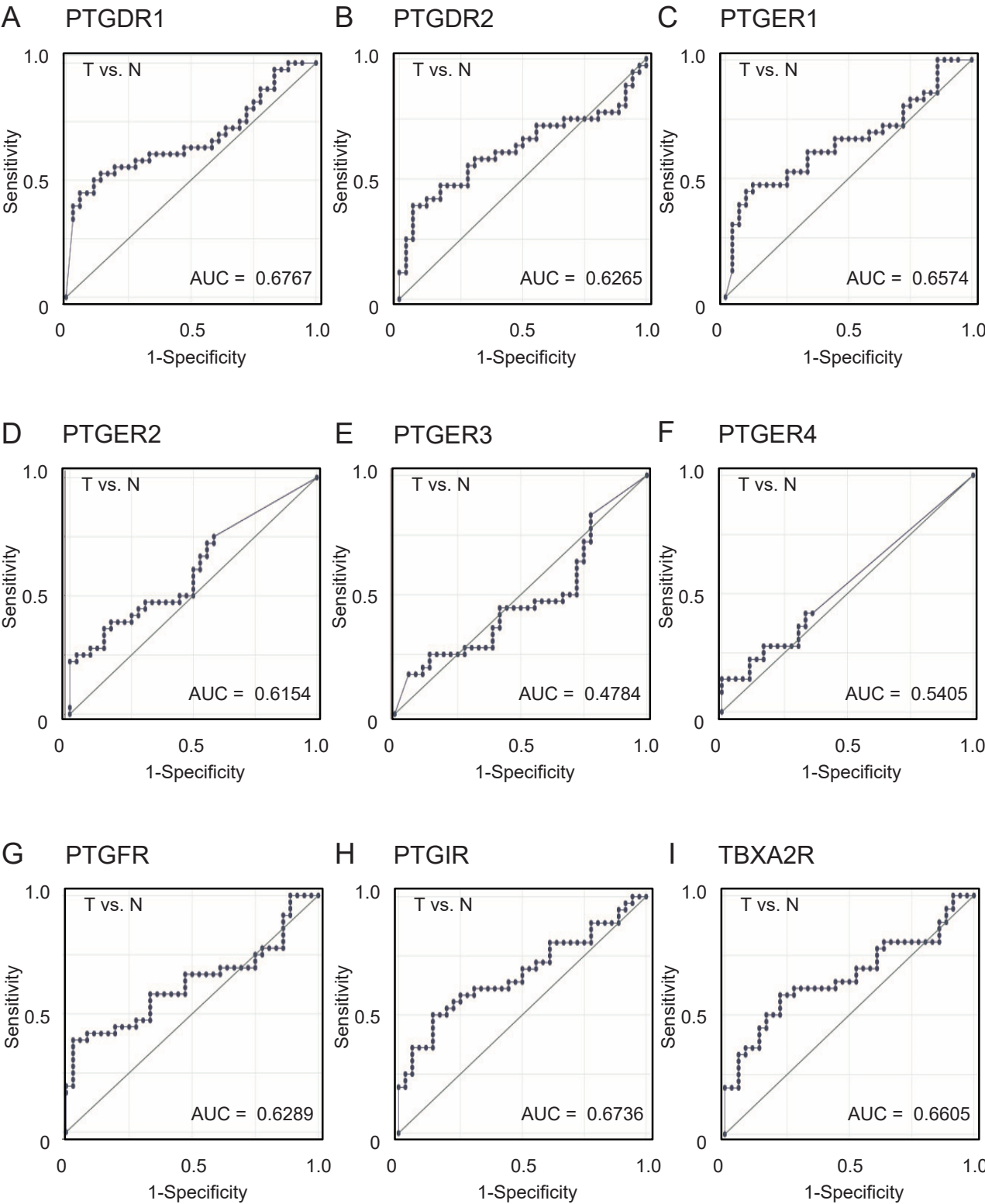

Supplement: Supplementary file 5 — Additional file 5: Fig. S2. Receiver operating characteristic (ROC) curves for the methylation markers in cancer tissue versus adjacent normal mucosal tissue. Based on the ROC curve analysis, Area Under Curves (AUCs) are 0.6767 for PTGDR1 (A), 0.6265 for PTGDR2 (B), 0.6574 for PTGER1 (C), 0.6154 for PTGER2 (D), 0.4784 for PTGER3 (E), 0.5405 for PTGER4 (F), 0.6289 for PTGFR (G), 0.6736 for PTGIR (H) and 0.6605 for TBXA2R (I). [file 12967_2020_2214_MOESM5_ESM.pdf]

Figure S3 Kiyoshi Misawa

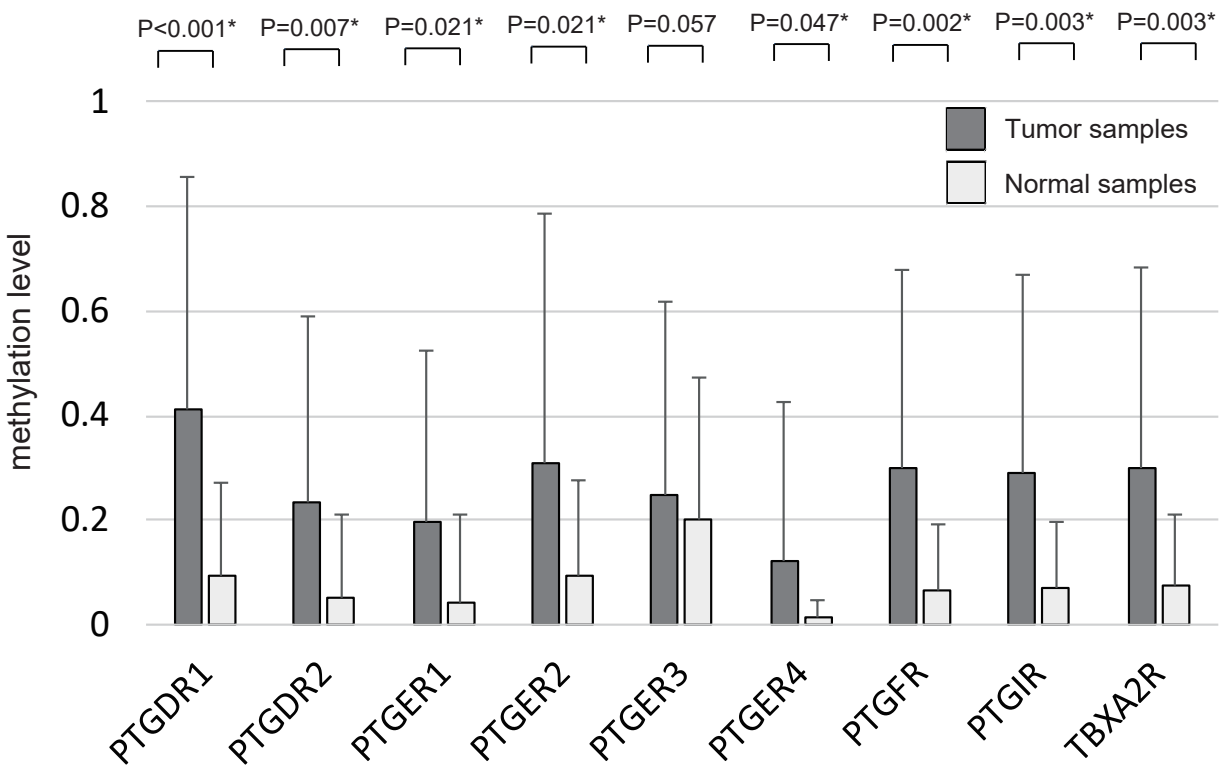

Supplement: Supplementary file 6 — Additional file 6: Fig. S3. Hypermethylation patterns in 36 matched pairs of head and neck tumors and adjacent normal mucosal tissues. The NMVs for the PTGDR1 (A), PTGDR2 (B), PTGER1 (C), PTGER2 (D), PTGER3 (E), PTGER4 (F), PTGFR (G), PTGIR (H) and TBXA2R (I) promoters were significantly higher in head and neck tumor tissues (T) than in paired adjacent normal mucosal tissue (N). The differences were significant as determined by the Student’s t‑test. *P < 0.05. [file 12967_2020_2214_MOESM6_ESM.pdf]

Figure S4 Kiyoshi Misawa

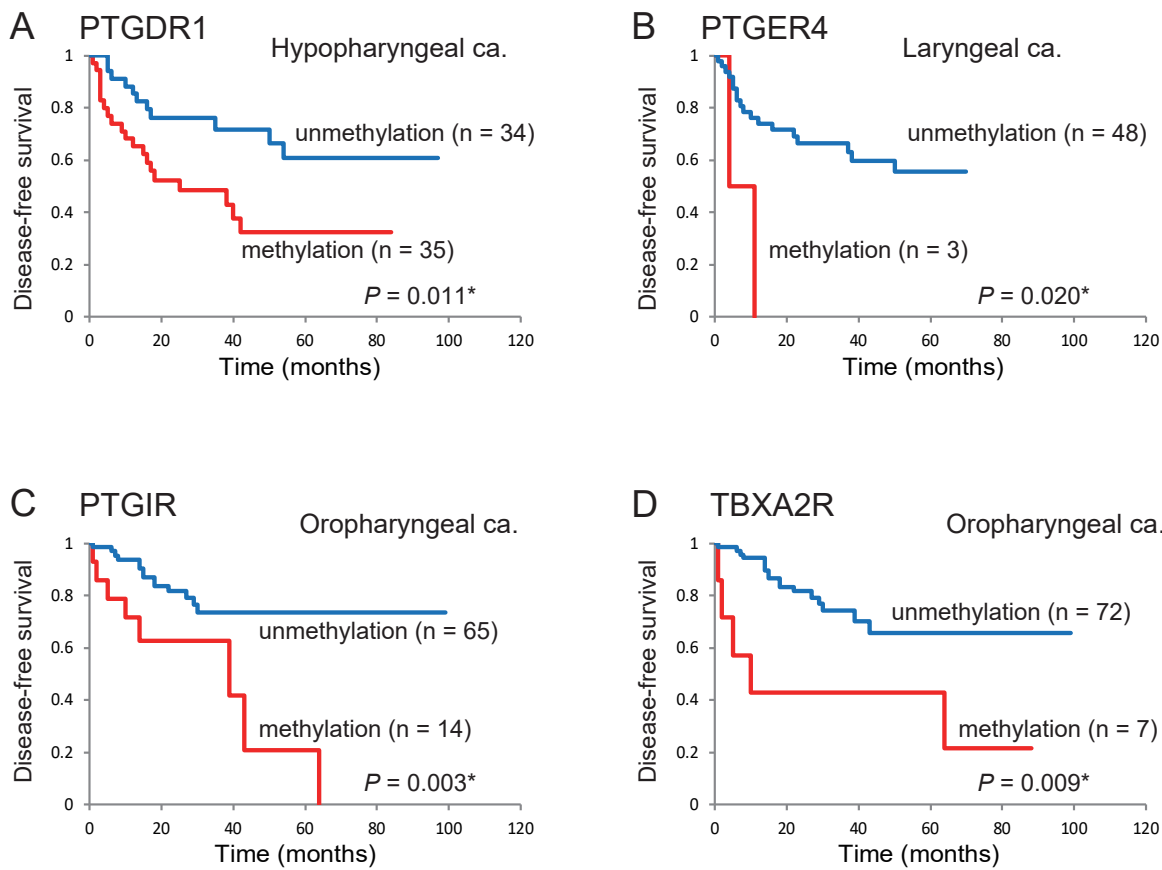

Supplement: Supplementary file 8 — Additional file 8: Fig. S4. Kaplan–Meier survival curves. Kaplan–Meier survival curves for PTGDR1 in (A) patients with hypopharyngeal cancer (n = 69), for PTGER4 in (B) patients with laryngeal cancer (n = 51), and for PTGIR and TBXA2R in (C and D) patients with oropharyngeal cancer (n = 79). The log-rank test was used to compare the survival times between patients with methylated (red lines) and unmethylated (blue lines) genes. *P < 0.05. [file 12967_2020_2214_MOESM8_ESM.pdf]

Figure S5 Kiyoshi Misawa

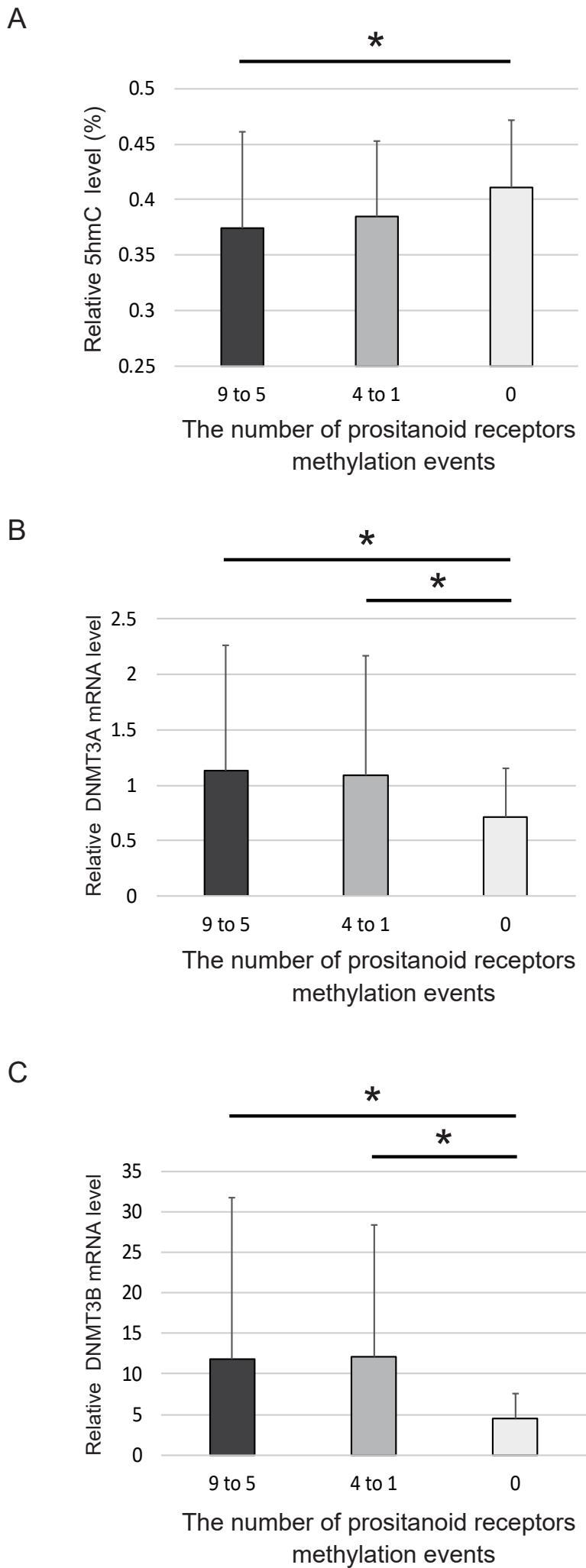

Supplement: Supplementary file 9 — Additional file 9: Fig. S5. Comparison of methylation frequencies between nine prostanoid receptor genes and other epigenetic factors. (A) 5hmC levels, (B) DNMT3A mRNA levels, (C) DNMT3B mRNA levels. *P < 0.05. The data are shown as the mean ± SE. [file 12967_2020_2214_MOESM9_ESM.pdf]

Figure S6 Kiyoshi Misawa

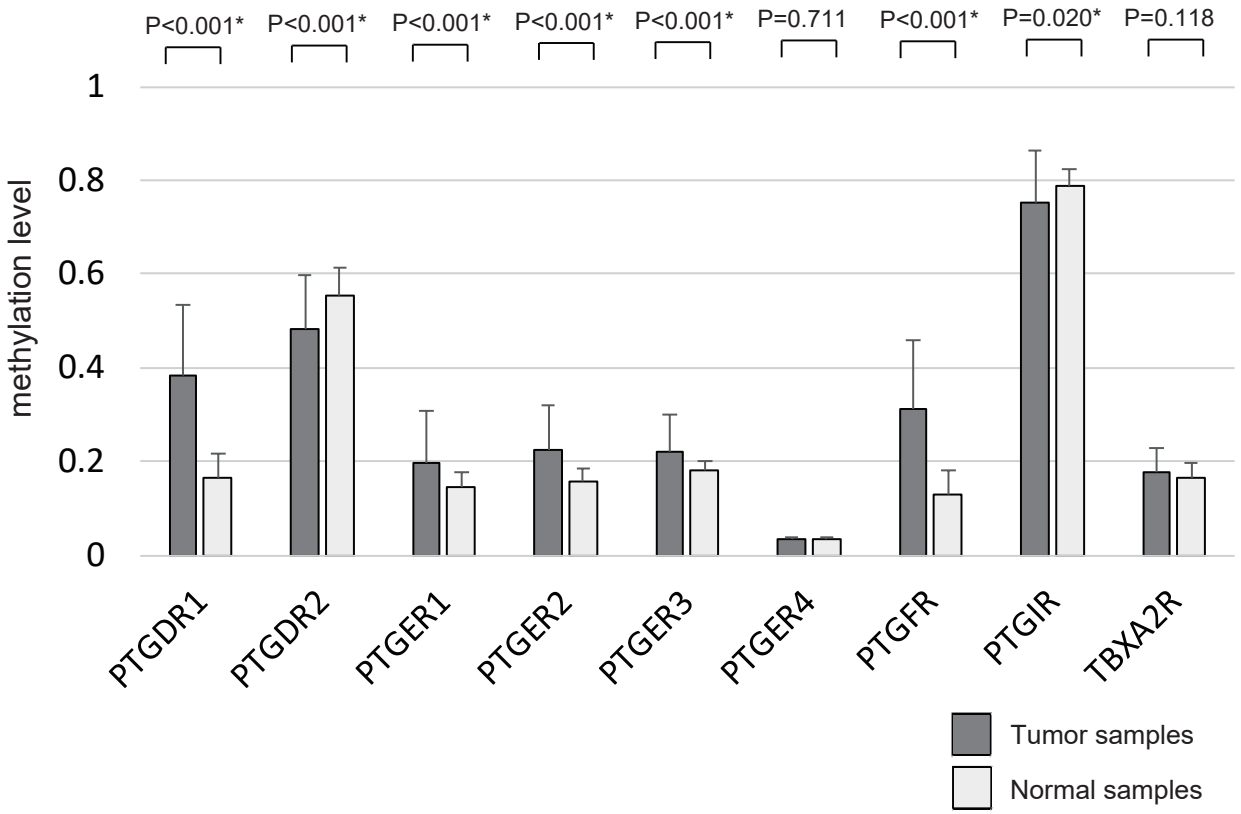

Supplement: Supplementary file 10 — Additional file 10: Fig. S6. Methylation status of the five neuropeptide receptor genes in HNSCC and normal samples in TCGA database. The methylation data for PTGDR1, PTGDR2, PTGER1, PTGER2, PTGER3, PTGER4, PTGFR, PTGIR and TBXA2R in HNSCC and normal samples were collected from TCGA database. *P < 0.05. [file 12967_2020_2214_MOESM10_ESM.pdf]
