# Supplementary material for: Prostanoid receptor genes confer poor prognosis in head and neck squamous cell carcinoma via epigenetic inactivation
Source: J Transl Med. 2020 Jan 21;18:31. doi: 10.1186/s12967-020-02214-1 (PMC6977280; doi:10.1186/s12967-020-02214-1)
Supplement: Supplementary file 4 — Additional file 4: Table S3. Results of the ROC curve analysis, the sensitivity, specificity, and cutoff value. [file 12967_2020_2214_MOESM4_ESM.pdf]

Additional file 4: Table S3. Results of the ROC curve analysis, the sensitivity, specificity, and cutoff value.

Genes	ROC Area	Sensitivity (%)	Specificity (%)	Cutoff value
PTGDR1	0.6767	86.11	69.44	0.1609
PTGDR2	0.6265	94.44	66.67	0.1231
PTGER1	0.6574	88.89	68.06	0.0484
PTGER2	0.6154	83.33	61.11	0.1614
PTGER3	0.4769	86.11	55.56	0.5023
PTGER4	0.5405	100.00	56.94	0.4192
PTGFR	0.6289	97.22	68.06	0.3681
PTGIR	0.6736	86.11	68.06	0.1089
TBXA2R	0.6605	77.78	68.06	0.0823
