# Supplementary material for: Prostanoid receptor genes confer poor prognosis in head and neck squamous cell carcinoma via epigenetic inactivation
Source: J Transl Med. 2020 Jan 21;18:31. doi: 10.1186/s12967-020-02214-1 (PMC6977280; doi:10.1186/s12967-020-02214-1)
Supplement: Supplementary file 7 — Additional file 7: Table S4. Results of log-rank tests for effect of number of methylated genes on disease free survival in 274 HNSCC. [file 12967_2020_2214_MOESM7_ESM.pdf]

**Additional file 7: Table S4. Results of log-rank tests for effect of number of methylated genes on disease free survival in 274 HNSCC.**

| No. methylated genes | No. patients with profile | P |
|---|---|---|
| ≥0 | 274 | |
| ≥1 | 154 | 0.130 |
| ≥2 | 125 | 0.187 |
| ≥3 | 79 | 0.228 |
| ≥4 | 62 | 0.090 |
| ≥5 | 50 | 0.007* |
| ≥6 | 41 | 0.036* |
| ≥7 | 30 | 0.103 |
| ≥8 | 18 | 0.940 |
| ≥9 | 4 | 0.931 |
