# Supplementary material for: Prostanoid receptor genes confer poor prognosis in head and neck squamous cell carcinoma via epigenetic inactivation
Source: J Transl Med. 2020 Jan 21;18:31. doi: 10.1186/s12967-020-02214-1 (PMC6977280; doi:10.1186/s12967-020-02214-1)
Supplement: Supplementary file 11 — Additional file 11: Table S5. Distribution of expression levels in TCGA cohort of HNSCC. [file 12967_2020_2214_MOESM11_ESM.pdf]

Additional file 11: Table S5. Distribution of expression levels in TCGA cohort of HNSCC

	IL-6 High expression (n=248)	IL-6 Low expression (n=249)	P-value	IL-11 High expression (n=248)	IL-11 Low expression (n=249)	P-value	RANKL High expression (n=248)	RANKL Low expression (n=249)	P-value
PTGDR1 mRNA level ± SD	16.78±12.21	8.87±8.60	< 0.001*	14.46±12.51	11.19±9.60	0.001*	17.18±13.07	8.50±6.75	< 0.001*
PTGER1 mRNA level ± SD	3.90±7.48	6.23±20.38	0.093	5.73±19.92	4.49±8.95	0.368	2.69±5.08	7.51±20.92	< 0.001*
PTGER2 mRNA level ± SD	48.49±64.63	18.10±24.68	< 0.001*	36.01±64.84	30.38±31.77	0.218	44.77±64.52	21.69±28.30	< 0.001*
PTGER3 mRNA level ± SD	96.53±112.4	64.78±151.0	0.008*	72.83±92.44	88.00±165.0	0.207	103.5±160.4	57.54±96.00	< 0.001*
PTGER4 mRNA level ± SD	308.6±220.6	194.9±177.6	< 0.001*	245.0±226.9	256.9±187.4	0.524	299.0±218.5	203.3±185.3	< 0.001*
PTGFR mRNA level ± SD	86.71±178.5	19.55±25.38	< 0.001*	58.33±170.9	47.44±73.06	0.355	77.05±177.4	28.87±44.59	< 0.001*
PTGFIR mRNA level ± SD	66.47±58.85	46.71±95.01	0.006*	57.64±53.68	55.29±98.93	0.743	72.63±90.17	40.42±63.75	< 0.001*
TBXA2R mRNA level ± SD	43.55±36.18	30.26±26.04	< 0.001*	43.54±38.72	30.19±22.00	< 0.001*	44.48±32.40	29.26±30.06	< 0.001*

Student t test * P < 0.05 Data are expressed as means ± standard deviation.
